# Aberrant Proliferation of Differentiating Alveolar Cells Induces Hyperplasia in Resting Mammary Glands of SV40-TAg Transgenic Mice

**DOI:** 10.3389/fonc.2014.00168

**Published:** 2014-06-26

**Authors:** Timo Quante, Florian Wegwitz, Julia Abe, Alessandra Rossi, Wolfgang Deppert, Wolfgang Bohn

**Affiliations:** ^1^Heinrich Pette Institute, Leibniz Institute for Experimental Virology, Hamburg, Germany; ^2^Institute for Tumor Biology, University Hospital Eppendorf, Hamburg, Germany

**Keywords:** SV40-TAg, hyperplasia, alveologenesis, mammary gland, tumorigenesis

## Abstract

WAP-T1 transgenic mice express SV40-TAg under control of the whey acidic protein (WAP) promoter, which directs activity of this strong viral oncogene to luminal cells of the mammary gland. Resting uniparous WAP-T1 glands develop hyperplasia composed of TAg positive cells prior to appearance of advanced tumor stages. We show that cells in hyperplasia display markers of alveolar differentiation, suggesting that TAg targets differentiating cells of the alveolar compartment. The glands show significant expression of *Elf5* and milk genes (*Lalba*, *Csn2*, and W*ap*). TAg expressing cells largely co-stain with antibodies to Elf5, lack the epithelial marker Sca1, and are hormone receptor negative. High expression levels of *Elf5* but not of milk genes are also seen in resting glands of normal BALB/c mice. This indicates that expression of Elf5 in resting WAP-T1 glands is not specifically induced by TAg. CK6a positive luminal cells lack TAg. These cells co-express the markers prominin-1, CK6a, and Sca1, and are positive for hormone receptors. These hormone sensitive cells localize to ducts and seem not to be targeted by TAg. Despite reaching an advanced stage in alveolar differentiation, the cells in hyperplasia do not exit the cell cycle. Thus, expression of TAg in conjunction with regular morphogenetic processes of alveologenesis seem to provide the basis for a hormone independent, unscheduled proliferation of differentiating cells in resting glands of WAP-T1 transgenic mice, leading to the formation of hyperplastic lesions.

## Introduction

Breast cancer, a progressive disease of mammary gland epithelia, is marked by consecutive appearance of different alterations, namely hyperplastic lesions, carcinoma *in situ*, and invasive, malignant adenocarcinomas. The mutational signature of human breast cancer supports the view of a direct evolutionary relationship between pre-neoplastic and malignant lesions (1–5). Mutations that inactivate functions of tumor suppressor proteins and thereby decrease genomic stability seem to be basic to initiation of breast cancer. Transgenic mouse strains were developed to mimic this process at an experimental level and to decipher the mechanisms initiating breast cancer and promoting progression to malignant stages. Major questions concern the cell types targeted by oncogenes in these models as this might determine the phenotype of tumor cells appearing at advanced stages. Here, we asked whether non-malignant, hyperplastic lesions appearing in resting glands of WAP-T1 transgenic mice (6) reflect selection of a distinct epithelial cell type or whether these lesions already display a heterogeneous cell composition as is seen in advanced tumor stages.

WAP-T1 mice express SV40-T-antigen (TAg) under control of the whey acidic protein (WAP) promoter, which directs activity of this strong viral oncogene specifically to epithelial cells of the gland. The transgene encodes both, the large (LT) and small (t) T-antigen. They cooperate to inactivate the tumor suppressor proteins RB and p53, alter expression of cell cycle regulating genes, promote unscheduled transition through G1/S, and impair DNA-damage response mechanisms, events that are known to decrease genomic stability (7–9).

The WAP promoter responds to lactotrophic hormones and thus is inactive in glands of virgin mice, active during lactation in differentiated luminal epithelial cells of the lobulo-alveolar complex, and at a lower level also active in the resting gland during estrous cycle (10–14). In accordance, epithelial cells of glands in virgin mice lack TAg whereas differentiated epithelial cells of the lactating gland show high levels of this protein. TAg positive cells of the lactating gland disappear with regression of lobulo-alveolar structures during involution (15–17). Sections of resting glands derived from uniparous mice show re-appearance of T-antigen positive epithelial cells at about 30 days post-weaning (p.w.). They constitute small hyperplastic lesions, which gradually increase in size and completely pervade the gland at 100 days p.w. Similar to human breast cancer, these hyperplastic lesions develop prior to appearance of carcinoma *in situ* and advanced tumor stages WAP-T1 (2–6). Malignant tumors develop late at low frequency in glands of WAP-T1 mice. They reveal a gene expression profile that recapitulates the phenotype of aggressive human cancers (18).

The data suggest that carcinogenesis in resting glands of WAP-T1 is largely delayed or halted at the stage of hyperplastic lesions. The cellular composition of hyperplasia and the status of TAg expressing epithelial cells in these lesions compared to lactating glands and advanced tumor stages are not defined. It was speculated that TAg expression in WAP-T1 selects for certain epithelial cell types. A gene expression analysis showed that WAP-T1 tumor samples are enriched in transcription factors relevant for embryonic stem cell maintenance. It led one to assume that TAg expression may favor survival and proliferation of cells displaying features of epithelial stem or progenitor cells (19). But not only stem or progenitor cells but also cells at advanced stages of differentiation have been proposed to generate hyperplastic lesions in transgenic mouse models (20–24).

Epithelia of the mouse mammary gland reveal a complex composition, marked by stem and progenitor cells, terminally differentiated cells, and regulatory units, such as hormone sensing cells (25). They rapidly change composition and functional status of the layer in dependence of developmental stages and environmental signals. This raises the question whether oncogenic activity of TAg in WAP-T1 mice at the early stage of hyperplasia randomly targets epithelial cells or promotes selection of a distinct cell type. Gene expression analysis of advanced WAP-T1 tumors identified at least two different tumor entities, which completely differ in marker expression: (i) low grade tumors, exhibiting a basal-like and morphologically differentiated phenotype with loss of chromosomes 2 and 19 and (ii) high grade tumors marked by strong expression of the *Met* gene and by co-expression of keratin 8/18, keratin 6, and the mesenchymal marker vimentin (26). But, a heterogeneous cell composition of advanced tumors does not necessarily contradict the idea that TAg selects for a distinct epithelial cell type. Data obtained with a tumor cell line derived from WAP-T1 glands showed that tumor cells are equipped with phenotypic plasticity, which for instance allows these cells to acquire a mesenchymal or an epithelial phenotype depending on the tumor environment (27).

Our data show that hyperplasia in resting glands of WAP-T1 mice are uniformly composed of cells differentiating along the alveolar lineage. The results suggest that expression of the viral oncogene in luminal epithelial cells pre-disposed to alveologenesis induces unscheduled proliferation of differentiating cells and thereby causes formation of hyperplasia.

## Materials and Methods

### Mice

Inbred BALB/c and the transgenic WAP-SV40 early region mouse line T1 (6) were housed under SPF conditions in accordance with official regulations for care and use of laboratory animals (UKCCCR Guidelines for the Welfare of Animals in Experimental Neoplasia) and approved by Hamburg’s Authority for Health (Nr. 24/96).

### Preparation of mouse mammary glands and isolation of luminal cell subpopulations

Mammary glands were collected at indicated time points from virgin mice, lactating mice, and uniparous mice of the BALB/c or WAP-T1 strains, respectively. Lymph nodes and tumors sometimes present at late stages in WAP-T1 mice were removed. Mammary glands to be used for RNA extraction were snap-frozen in liquid nitrogen. Glands intended for immunofluorescence were embedded in Shandon Cryomatrix (Thermo Scientific) and frozen at −80°C. To extract cells for subsequent FACS-sorting all mammary glands from one mouse were pooled in L15 Medium (Sigma-Aldrich), transferred to a sterile Petri dish and minced with scalpels. The organoid suspension was digested in serum-free L15 Medium with 3 mg/ml Collagenase Type I (Life Technologies) and 1.5 mg/ml trypsin (Sigma-Aldrich) for 1 h at 37°C. Cells were collected by centrifugation at 300 × *g* for 5 min and washed once in L15 + 10% fetal calf serum (FCS). Red blood cells were lysed by two rounds of incubation with red blood cell lysis buffer (Sigma-Aldrich) and after two washes with PBS/0.02% EDTA cells were incubated for 15 min at 37°C in SMEM Medium (Life Technologies). Cells were collected by centrifugation and incubated for 2 min at 37°C in 2 ml of 0.25% trypsin/0.2% EDTA in HANKS balanced salt solution (Sigma-Aldrich); Cells were resuspended in 5 ml L15 medium, 400 U/ml Dnase I (Sigma-Aldrich) were added, and the cells were incubated for another 5 min at 37°C before the reaction was stopped by addition of 5 ml L15 + 10% FCS. To obtain a single cell suspension, the cells were passed through a 30 μm filter (Miltenyi Biotech). Lineage depletion was performed using a mouse Lineage Cell Depletion Kit (Miltenyi Biotech), which selects for CD5, CD45R (B220), CD11b, Gr-1 (Ly-6G/C), 7-4, and Ter-119 positive cells and permits subsequent isolation of Sca1 positive cells.

### FACS-sorting

After lineage depletion cells extracted from mammary glands were stained with FITC hamster anti-mouse CD61 (BD Biosciences; 1 μg/10^6^ cells), PE hamster anti-mouse/rat CD29 (BioLegend; 0.4 μg/10^6^ cells), and AlexaFluor647 rat anti-mouse Ly-6A/E (Sca1) (BioLegend; 0.5 μg/10^6^ cells). Respective isotype controls were included. The cells were sorted on a FACS Aria (BD Biosciences). After sorting, aliquots of cells for immunofluorescence were plated on poly-lysine coated cover slips. Cells for RNA extraction were collected by centrifugation.

Sizes of subpopulations were derived from independent FACS experiments (BALB/c *n* = 10; WAP-T1 *n* = 23). In each experiment, the percentage of total cells was determined for each subpopulation and mean values ± SEM were calculated from all experiments. Statistical significance was assessed using Student’s *t*-test and *p*-values <0.05 were considered as statistically significant.

### RNA extraction and quantitative real time PCR

Total RNA was purified using peqGOLD TriFast (Peqlab) according to the manufacturer’s protocol. FACS-sorted cells were lysed directly in TRIfast, frozen mouse tissue was carefully cut into small pieces on dry ice, transferred to a lysing matrix tube (MP Biomedicals), and lysed in TRIfast by two cycles of 5 s in a FastPrep instrument (MP Biomedicals). DNA was removed by DNAse I digestion (QIAGEN) followed by another round of RNA extraction using TRIfast. Reverse transcription was performed with the High Capacity RT kit (Applied Biosystems) according to the manufacturer’s protocol. PCR was performed using the Power SYBR Green PCR Master Mix (Applied Biosystems) in a standard program (10 min 95°C; 15 s 95°C, 1 min 60°C; 40 cycles) running in an ABI 7900HT Fast Thermal Cycler (Applied Biosystems). PCR reactions for each sample were repeated in triplicates. The integrity of the amplified products was confirmed by melting-curve analysis. PCR primers (Table S1 in Supplementary Material) were designed using the Primer3 web tool (http://biotools.umassmed.edu/bioapps/primer3_www.cgi). PCR efficiency was measured for each primer pair using serial dilutions of cDNA. 18S rRNA or HSC70 were used as endogenous controls and relative quantitation of transcript levels was performed based on the 2^−ddCt^ method.

For the gene expression analysis in whole mammary glands (Figure 5), 10 BALB/c and 10 WAP-T1 mammary glands from five mice each were analyzed and fold changes relative to a BALB/c mammary gland were calculated. Mean values and SEM were calculated from the 10 single values for BALB/c and WAP-T1 mice. Statistical significance was assessed using Student’s *t*-test and *p*-values <0.05 were considered statistically significant.

For the gene expression analysis in sorted subpopulations (Figures 6C, 7 and 9) data was derived from at least three independent sorting experiments (Figure 6C: BALB/c and T1 *n* = 5; Figures 7 and 9: BALB/c *n* = 4, T1 *n* = 5, BALB/c vir and T1 vir *n* = 3). Fold changes relative to BALB/c subpopulations (Figure 6C: CD29hi; Figures 7 and 9: CD61^+^Sca^+^) were calculated and data from single experiments were summarized as mean values ± SEM. Statistical significance was assessed using Student’s *t*-test and *p*-values <0.05 were considered as statistically significant.

### Immunohistochemistry

Tissue specimens were fixed overnight with 4% formaldehyde and 1% acetic acid, and stored in 4% formaldehyde at 4°C. Fixed tissue was embedded in Paraplast X-TRA (Sherwood Medical). Deparaffinated sections were stained with H&E (Sigma) according to standard laboratory protocols. For labeling with antibodies deparaffinated sections were treated with an antigen retrieval solution (Citra Plus, Biogenex). Sections were blocked with normal serum for 1 h at room temperature and stained with primary antibodies at appropriate dilutions overnight at 4°C. Bound antibodies were detected using alkaline phosphatase-, respectively peroxidase-labeled anti Ig detection systems (Envision, DakoCytomation). Alkaline phospatase activity was visualized using naphthol AS-BI-phosphate and New Fuchsin (Fuchsin plus substrate-chromogene, DakoCytomation) as substrate; peroxidase activity was identified using diaminobenzidine (DAB) as substrate. Sections were counterstained with hemalum and embedded in Mowiol. Photographs were taken with a LEICA DMI6000B.

For preparation of cryosections glands embedded in cryomatrix were sectioned in a LEICA CM 3050 cryotome at −30°C. Sections were attached to glass cover slips and stored at −20°C. Sections attached to glass coverslips were incubated with −20°C acetone for 10 min, acetone was evaporated at room temperature for 5 min, and sections were rehydrated in PBS for 10 min at room temperature. Staining was done at room temperature in a dark chamber. Sections were blocked with 5% normal serum for 45 min, incubated with the primary antibody for 1 h, washed with PBS three times for 10 min each, stained with fluorochrome labeled secondary antibody for 1 h, and washed again with PBS three times 10 min each. Nuclei were visualized by counterstaining with DAPI or DRAQ5. Stained sections were embedded in Mowiol. Photographs were taken with a Zeiss LSM 501 confocal microscope or with epifluorescence microscopes (LEICA DMI6000B and LEICA DMRA).

### Antibodies

Primary antibodies directed to SV40-TAg were derived from guinea pig or rabbit. Commercial antibodies used were CK6a (PRB-169P-100, Covance), CK8/18 (AcrisBP5007), CK14 (Acris BP5009), Mcm2 (N-19 sc-9839, Santa Cruz), Ki67 (H-300, sc-15402, Santa Cruz), ELF5 (N-20, sc9645, Santa Cruz), PR (C-18 sc-538, Santa Cruz), ER (M-20 sc542, Santa Cruz), CD 133 (clone 13A4, eBioscience 14-1331-82), Ly-6A/E (Sca1) (BioLegend), and phospho-histone H3 (Ser10) (Cell Signaling 9701). Antibodies conjugated with Alexa Fluor dyes (Alexa 488, Alexa 555, Alexa 633) (Molecular Probes) were used as secondary antibodies.

## Results

### Resting uniparous WAP-T1 glands show features of alveologenesis

**Figure 1:**
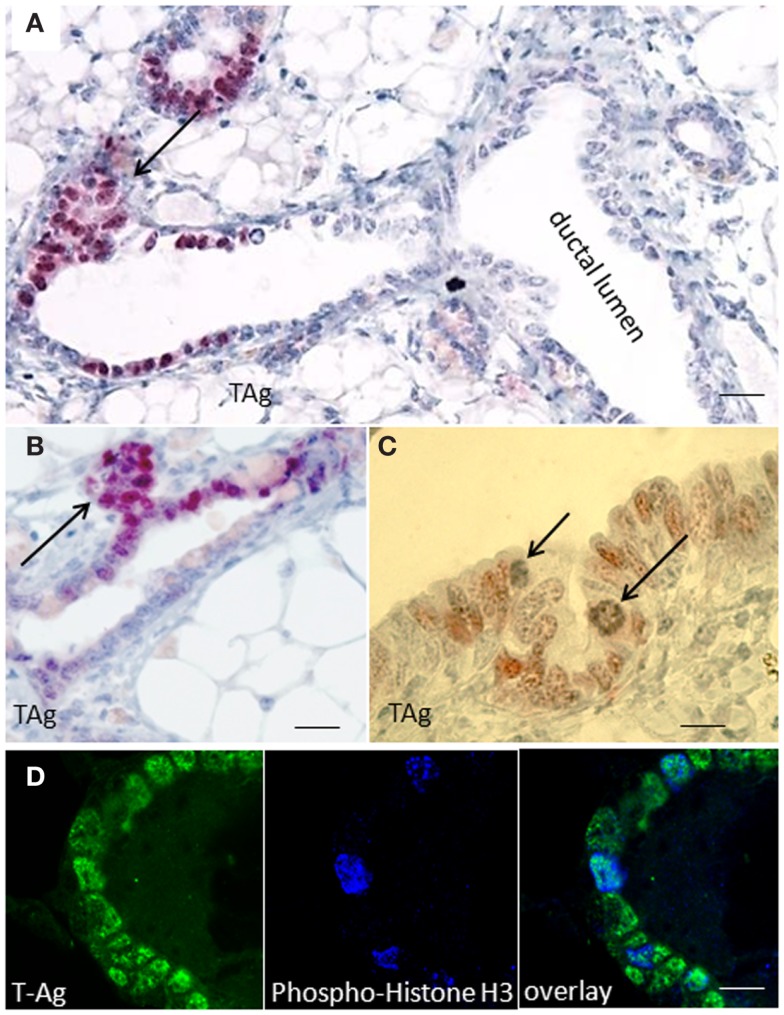
**SV40-TAg positive epithelial cells cluster at sites of bud formation**. **(A–C)** TAg labeling on sections of paraffin embedded WAP-T1 glands isolated 60 days post-weaning (p.w.); alkaline phosphatase **(A,B)** or peroxidase **(C)** conjugated antibodies were used as secondary antibodies; TAg expressing cells cluster at sites of bud formation [arrows in **(A,B)**]; individual TAg positive epithelial cells show condensing chromatin [arrows in **(C)**]; **(D)** IF double labeling with antibodies to TAg (green) and phospho-histone H3 (blue) on cryosections of resting uniparous WAP-T1 glands; phospho-histone H3 staining points to mitotic activity in WAP-T1 hyperplastic lesions. Bars: **(A)** = 50 μm; **(B)** = 15 μm; **(C,D) ** = 10 μm.

Immunohistochemical studies performed on paraffin sections of resting uniparous WAP-T1 glands showed local clusters of TAg positive luminal epithelial cells in ducts (Figure 1A). These clusters were often associated with processes of side bud formation (Figure 1B, arrow) Condensing chromatin in individual TAg positive cells (see Figure 1C, arrows) suggested mitotic activity in these lesions. Reaction of individual TAg positive cells with antibodies to phospho-histone H3, a marker of condensing chromosomes in mitosis, corroborates this suggestion (Figure 1D). The data suggest that TAg expressing cells are involved in morphogenetic processes inducing lobulo-alveolar structures.

**Figure 2:**
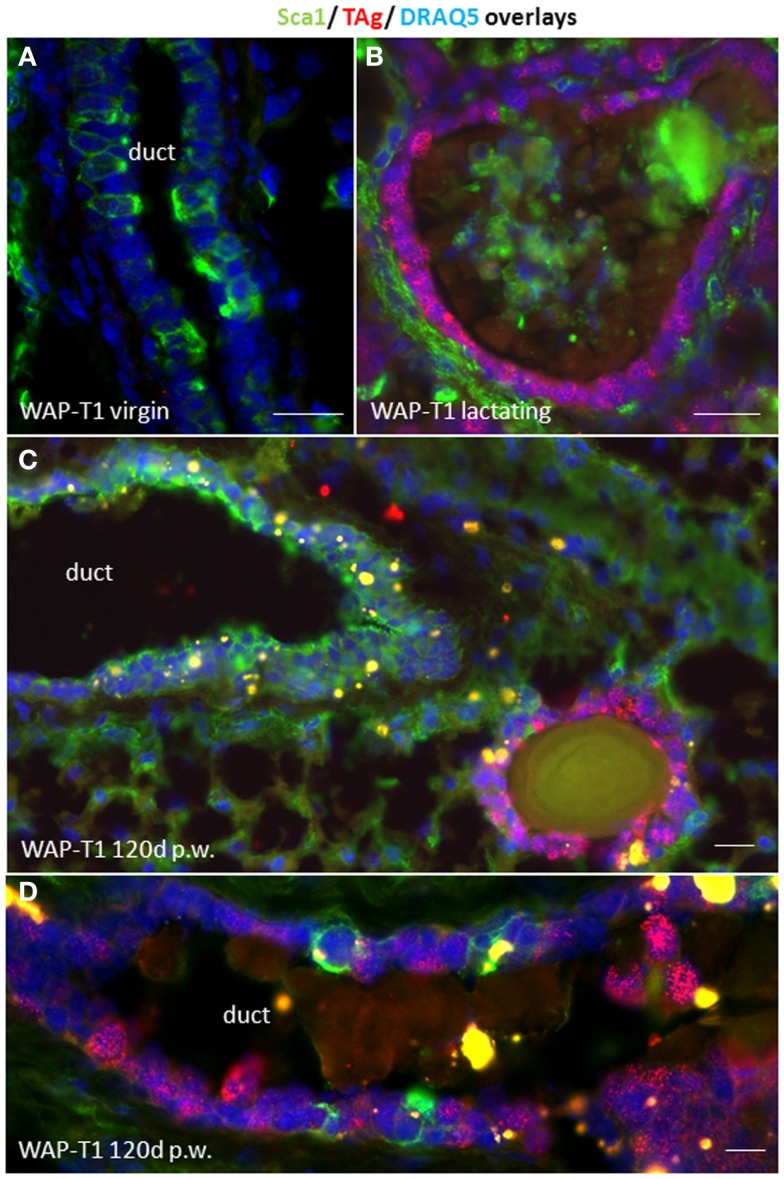
**TAg is expressed in Sca1 negative luminal epithelial cells**. **(A,B)** IF double labeling with antibodies to Sca1(green) and TAg (red) on cryosections of WAP-T1 glands; staining of DNA with DAPI (blue); **(A)** luminal epithelia in virgin WAP-T1 glands are TAg negative and reveal a heterogenous Sca1 staining pattern; **(B)** luminal epithelia in lactating WAP-T1 glands are TAg positive and Sca1 negative; **(C,D)** TAg positive cells in epithelial compartments of resting uniparous WAP-T1 glands (120 days p.w.) are also Sca1 negative. Bars: **(A–C)** = 50 μm;**(D)** = 5 μm.

To characterize TAg positive cells in relation to functional stages of luminal epithelia we performed an IF double labeling study with antibodies to Sca1 and SV40-TAg on cryosections of WAP-T1 glands. Sca1 is a GPI-anchored protein that was originally identified in hematopoietic stem cells. In the mouse mammary gland, it was reported to mark luminal epithelial cells in ducts and bipotent luminal progenitor cells of the lobulo-alveolar compartment, but not terminally differentiated alveolar cells (28–30). In our labeling study, we included glands from virgin mice, lactating mice, and uniparous mice (120 days p.w.). As shown in Figure 2A, luminal epithelia of virgin WAP-T1 glands contained Sca1 positive and negative cells, but were generally devoid of TAg. Luminal epithelia of lactating glands were TAg positive but Sca1 negative (Figure 2B). In similar, cells in hyperplastic lesions of resting uniparous WAP-T1 glands which stained positively for TAg were also Sca1 negative (Figures 2C,D). Sca1 positive epithelial cells in glands were confined to luminal epithelia of ducts and did not stain with antibodies to TAg. These findings suggest that TAg expression is confined to Sca1 negative luminal epithelial cells.

**Figure 3:**
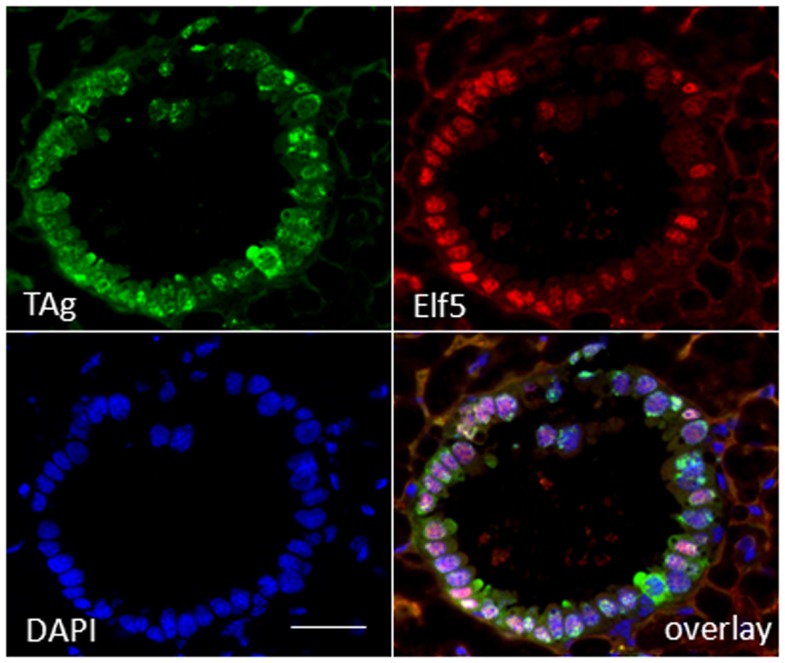
**TAg positive cells in hyperplasia co-stain with antibodies to ELF5**. IF double labeling on cryosections of resting uniparous WAP-T1 glands (120 days p.w.) with antibodies to TAg (green) and ELF5 (red); staining of DNA with DAPI; individual TAg positive cells lack ELF staining signal. Bar = 50 μm.

To substantiate a relationship of TAg expression with alveologenesis, we assayed resting uniparous WAP-T1 glands (120 days p.w.) for expression of ELF5, a transcription factor known as master regulator of alveologenesis (31). Immunofluorescence staining on cryosections demonstrated that TAg positive cells co-expressed ELF5 (Figure 3). But, expression of TAg and ELF5 in epithelia of hyperplasia did not totally overlap. A proportion of TAg positive cells showed only weak staining or was ELF5 negative (Figure 3).

The data suggest that TAg and Sca1 mark different functional entities of cells in luminal epithelia of WAP-T1 glands: TAg^+^/Sca1^−^ cells representing the differentiating lobulo-alveolar compartment, and TAg^−^/Sca1^+^ identifying cells of the ductal epithelium.

**Figure 4:**
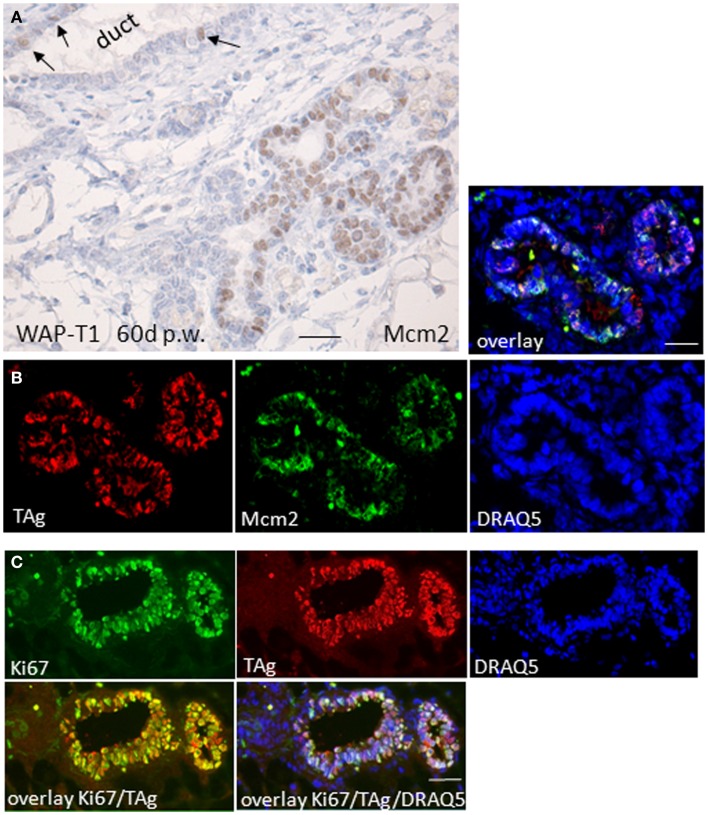
**TAg positive luminal epithelial cells co-stain with antibodies to proliferation markers**. **(A)** Immunoperoxidase labeling with Mcm2 antibodies on sections of paraffin embedded resting uniparous WAP-T1 glands (60 days p.w.) shows strong nuclear staining of epithelia in hyperplasia; in ductal epithelia only individual cells stain positively for Mcm2 (arrows); **(B,C)** IF double labeling on cryosections from resting uniparous WAP-T1 glands (120 days p.w.) shows coincident labeling of TAg (red) and Mcm2 (green) **(B)**, respectively TAg (red) and Ki67 (green) **(C)**; DNA staining with DRAQ5 (blue) Bars: **(A,B)** = 20 μm.

To determine whether TAg expressing cells in hyperplasia of WAP-T1 glands proliferate, we labeled cryosections with antibodies to Mcm2 and to Ki67, both of which are nuclear markers that are expressed in cycling cells. Mcm2 is a member of the licensing protein family, which facilitates coordinated transition from G1 into S-phase and is down-regulated, when cells exit the cell cycle (32). Human mammary gland epithelia show Mcm2 in nuclei of differentiating but not in terminally differentiated cells (33). Ki67 is expressed in G1, S, G2, and M-phase of the cell cycle but is absent from cells resting in G0. Thus, Ki67 is used to identify the proliferating cell compartment in tissue (34). Hyperplasia in resting uniparous WAP-T1 glands exhibited prominent Mcm2 staining as shown by immunoperoxidase labeling on paraffin sections (Figure 4A). IF double labeling on cryosections revealed coincident labeling of TAg and Mcm2 in epithelial cells (Figure 4B). TAg positive cells also co-stained with antibodies to Ki67 (Figure 4C). These results indicate that TAg expressing epithelial cells that accumulate in hyperplasia of resting WAP-T1 glands do not exit the cell cycle.

### Resting uniparous WAP-T1 glands show lactogenic activity

**Figure 5:**
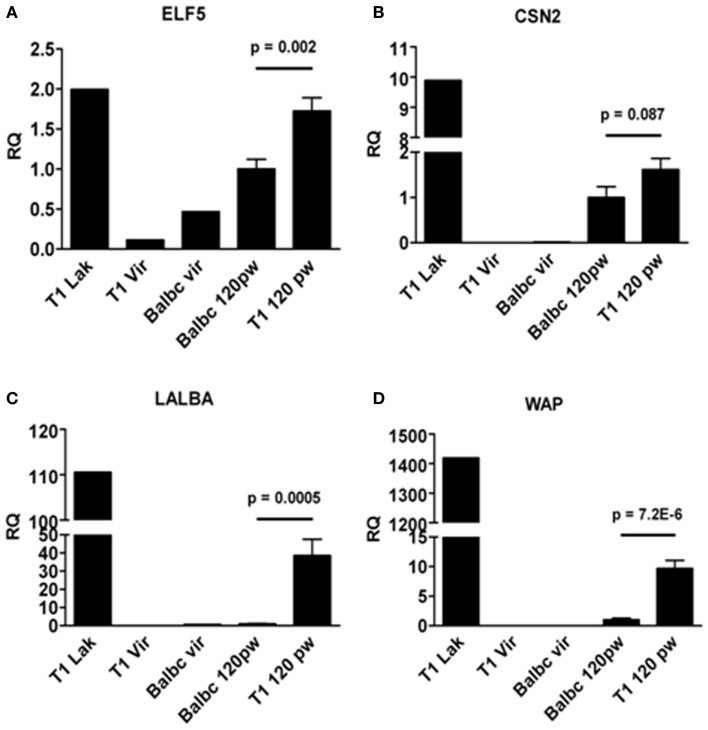
**qRT-PCR analysis of ELF5 and milk gene expression in whole glands**. *ELF5* **(A)** and milk genes (*CSN2*, *LALBA*, *WAP*) **(B–D)** are expressed in resting glands (120 days p.w.) from BALB/c and WAP-T1 mice (*n* = 10 from five mice); *ELF5*, **(A)**, *LALBA* **(C)**, and *WAP* **(D)** levels are significantly higher in WAP-T1 than in BALB/c glands; individual glands from lactating WAP-T1 mice (T1 Lak), WAP-T1 virgin mice (T1Vir), and BALB/c virgin mice (Balbc vir) are included for comparison; data presented as mean ± SEM.

Next, we asked whether formation of lobulo-alveolar structures in resting WAP-T1 glands correlated with lactogenic activity, which is marked by expression of the milk genes *WAP*, *Csn2* (beta-casein), and *Lalba* (lactalbumin). For comparison, we included in our analysis one gland from each, a lactating WAP-T1 mouse, a BALB/c virgin mouse, and a WAP-T1 virgin mouse. qRT-PCR analysis performed with whole glands (summarized in Figure 5) revealed high expression levels of *ELF5* and milk genes in glands of lactating WAP-T1 mouse (T1 Lak, Figures 5A–D), and low levels in glands from virgin mice (T1vir and BALB/c vir, Figures 5A–D). Resting glands of WAP-T1 (T1 120 pw) showed prominent *Elf5* expression (Figure 5A), clearly exceeding the level in resting BALB/c glands (BALBc 120 pw, Figure 5A) by 1.7-fold (*p* < 0.01). Milk gene expression was detectable in all glands, but showed clear differences between WAP-T1 and BALB/c. While *Csn2* expression was only slightly (~twofold, n.s.) upregulated in resting WAP-T1 glands compared to resting BALB/c glands (see BALB/c 120 pw and T1 120 pw in Figure 5B), *Lalba* and *Wap* expression levels were increased 40- and 10-fold, respectively (*p* < 0.001 and *p* < 1.00E-05) (Figures 5C,D). These data suggest enhanced alveologenesis in resting uniparous WAP-T1 glands.

### TAg expressing cells reach an advanced stage in alveolar differentiation

**Figure 6:**
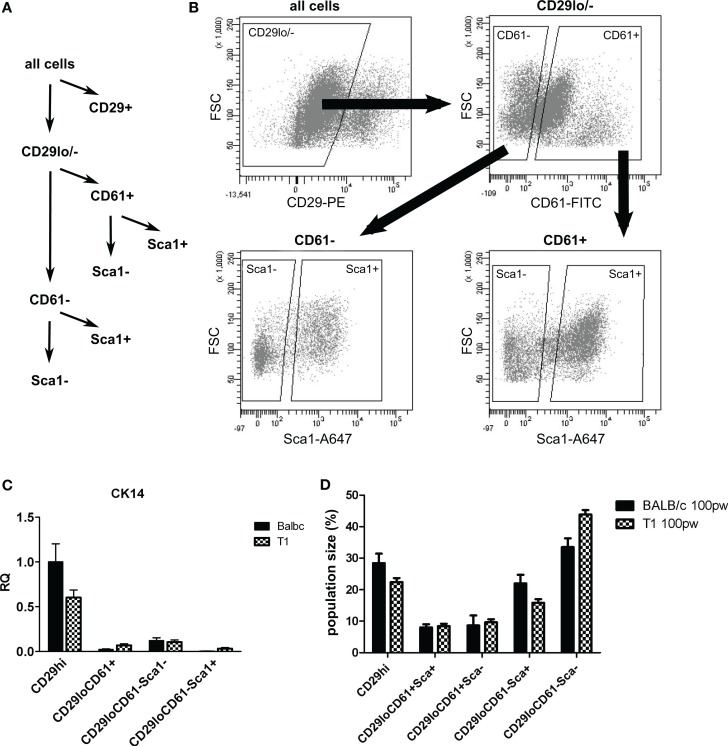
**Sorting strategy for isolation of luminal epithelial cell populations**. After lineage depletion, basal, myoepithelial cells (CD29^+^) were separated from luminal epithelial cells (CD29^lo/−^). Luminal epithelial cells were separated into undifferentiated (CD61^+^) and differentiated (CD61^−^) subpopulations and further separated into Sca1^−^ and Sca1^+^ subpopulations **(A,B)**. **(C)** CK14, a marker of myoepithelial cells, is enriched in the basal CD29hi subpopulation (*n* = 5). **(D)** Sizes of subpopulations from BALB/c (*n* = 10) and WAP-T1 (*n* = 23) mice as percent total cells; data presented as mean ± SEM. Population size of the CD61^−^/Sca1^+^ population was significantly decreased in WAP-T1 mice compared to BALB/c (15.6 vs. 27.9%, *p* < 0.05) while CD61^−^/Sca1^−^ population size was significantly increased (54.2 vs. 39.2%, *p* < 0.05).

Based on our *in situ* analysis, we asked if we could use Sca1 as marker to isolate luminal cell populations from WAP-T1 glands in order to define their status by RT-PCR. Single cell populations obtained after proteolytic digestion of glands and lineage depletion were fractionated into subpopulations applying fluorescent activated cell sorting. A first approach, trying to isolate the bulk of epithelial cells directly by use of CD24 did not reveal clear subpopulations in samples from WAP-T1 glands. Thus, we selected CD29 as marker to separate luminal (CD29^low^) from basal epithelial cell populations (CD29^high^). The CD29^low^ population was then fractionated into Sca1^+^ and Sca1^−^ subpopulations. In addition, we included CD61 as marker in our sorting strategy (Figure 6A). CD61 was described to mark luminal progenitor cells, endowed with the potential to differentiate into alveolar or ductal cells (35).Our sorting strategy is similar to that previously described by Shehata et al. (36), who also used Sca1 to isolate luminal cell subpopulations. But in difference to our approach they isolated epithelial cells directly by use of Epcam and substituted CD61 for CD49b.

Sorting yielded four different luminal subpopulations (Figure 6B), which were assayed for gene expression by qRT-PCR: the subpopulations CD29^low^/CD61^+^/Sca1^+^ and CD29^low^/CD61^−^/Sca1^+^, which we expected to be enriched with TAg negative cells, and the subpopulations CD29^low^/CD61^+^/Sca1^−^and CD29^low^/CD61^−^/Sca1^−^, which we expected to be enriched with TAg positive cells differentiating along the alveolar lineage. qRT-PCR (Figure 6C) verified that cells expressing the myoepithelial marker *CK14* clearly separated with the CD29^hi^ subpopulation. Thus, basal epithelial cells were separated efficiently from luminal cell subpopulations in this approach. In accordance, immunofluorescence staining showed CK14 positive cells only in CD29^hi^ subpopulations (data not shown). The relative proportion of CD61^+^ cells in samples isolated from resting glands of BALB/c and WAP-T1 mice was rather identical (Figure 6D). But compared to BALB/c, the proportion of CD61^−^/Sca1^+^ cells was significantly decreased (15.6 vs. 27.9%, *p* < 0.05) and that of CD61^−^/Sca1^−^ cells was significantly increased (54.2 vs. 39.2%, *p* < 0.05) in WAP-T1 samples. This suggests a relative shift in population sizes to differentiating alveolar cells in WAP-T1 glands.

**Figure 7:**
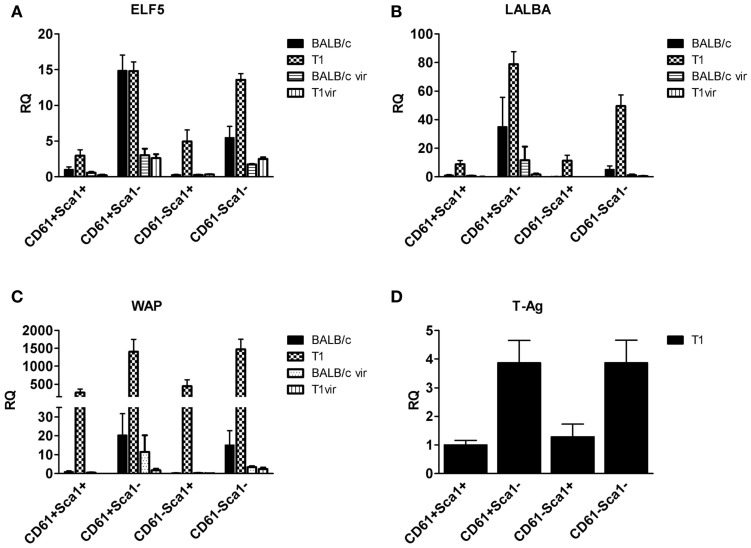
**qRT-PCR analysis of ELF5, milk gene (LALBA, WAP), and TAg expression in luminal epithelial cell subpopulations from resting (120 days p.w.) glands of uniparous mice (BALB/c, T1) and glands from virgin mice (BALB/c vir, T1 vir)**. ELF5 **(A)**, LALBA **(B)**, WAP **(C)**, and TAg **(D)** expression are most prominent in Sca1 negative cells; note equivalent ELF5 levels in undifferentiated (CD61^+^Sca1^−^) cells of BALB/c and T1, but different levels in differentiated (CD61^−^Sca1^−^) cells from both mouse strains; virgin mice show only basal expression of ELF5 and milk genes (BALB/c *n* = 4, T1 *n* = 5, BALB/c vir *n* = 3, T1 vir *n* = 3); ata presented as mean ± SEM.

qRT-PCR analysis of *TAg* expression in luminal cell subpopulations isolated from resting uniparous WAP-T1 glands (T1 in Figure 7D) showed high and nearly identical levels in the Sca1 negative subpopulations CD29^low^/CD61^+^/Sca1^−^(further named CD61^+^/Sca1^−^) and CD29^low^/CD61^−^/Sca1^−^(further named CD61^−^/Sca1^−^). *TAg* levels were fourfold lower in the Sca1 positive subpopulations CD29^low^/CD61^+^/Sca1^+^ (further named CD61^+^/Sca1^+^) and CD29^low^/CD61^−^/Sca1^+^ (further named CD61^−^/Sca1^+^). Sca1 negative subpopulations from resting WAP-T1 glands also displayed high expression levels of *Elf5*, *Wap*, and *Lalba* (T1 in Figures 7A–C). Expression levels of these genes were significantly lower in Sca1^+^ subpopulations.

Then, we asked if *ELF5* and milk gene expression observed in Sca^−^ subpopulations from resting WAP-T1 glands (T1 in Figures 7A–C) is also detectable in luminal cell subpopulations from resting BALB/c glands. qRT-PCR analysis revealed significant expression of *Elf5*, *Lalba*, and *Wap* in Sca1^−^ subpopulations from BALB/c but only basal expression levels in Sca1^+^ subpopulations (BALB/c in Figures 7A–C). Remarkably, *Elf5* expression (Figure 7A) in the Sca1^−^/CD61^+^ subpopulation reached the same high level as in WAP-T1, but was decreased 2.5-fold (*p* < 0.01) in the Sca1^−^/CD61^−^ subpopulation compared to WAP-T1. This indicates that Elf5 expression in differentiating alveolar cells of BALB/c does not reach the same high level as in WAP-T1. Overall expression levels of milk genes (Figures 7B,C) in Sca1^−^ subpopulations from BALB/c were low compared to WAP-T1: *Lalba* expression was reduced by ~twofold (n.s.) in CD61^+^/Sca1^−^ and ~10-fold (*p* < 0.01) in CD61^−^/Sca1^−^ subpopulations; *Wap* expression was reduced ~70- and 100-fold (*p* < 0.05 and *p* < 0.01), respectively.

The data indicate that alveolar differentiation marked by Elf5 also takes place in resting uniparous BALB/c glands and thus is not a specific feature of resting uniparous WAP-T1 glands. But, *ELF5* expression in subpopulations enriched with differentiated alveolar cells (CD61^−^/Sca1^−^) was significantly higher in WAP-T1 than in BALB/c mice. Furthermore, Sca1 negative subpopulations from WAP-T, but not from BALB/c showed significant expression of milk genes. This suggests that alveolar cells in resting uniparous WAP-T1 glands (120 days p.w.) reach a more advanced stage of differentiation marked by enhanced lactogenic activity.

### TAg does not target CK6a positive luminal epithelial cells

Luminal epithelia of the mouse mammary gland contain CK6a positive cells, which were repeatedly discussed to represent putative progenitor cells of the ductal and alveolar lineage (20, 37). Furthermore, they were described as potential targets of activated oncogenes in transgenic mice giving rise to mammary gland tumors (22, 23). High grade tumors in WAP-T1 mice showed significant expression of *CK6* as assayed by gene expression analysis (26). Thus, we asked whether TAg targets CK6a positive cells in resting uniparous WAP-T1 glands.

**Figure 8:**
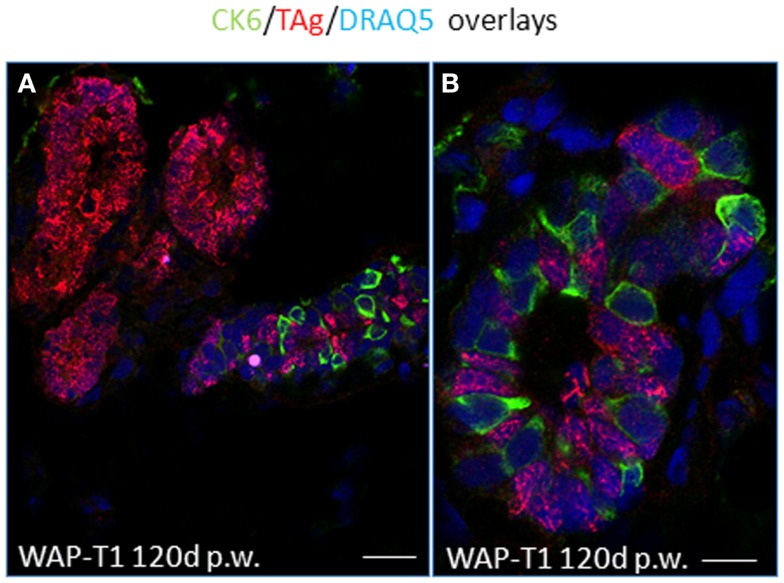
**TAg does not target CK6a positive luminal epithelial cells in resting uniparous WAP-T1 glands**. IF double labeling with antibodies to CK6a (green) and TAg (red) on cryosections from resting uniparous WAP-T1 glands (120 days p.w.); DNA staining with DRAQ5 (blue); TAg positive lesions lack CK6a positive cells **(A)**; CK6a and TAg mark different luminal epithelial cells in ductal compartments **(A,B)**. Bars: **(A)** = 30 μm;**(B)** = 5 μm.

IF studies on cryosections of resting uniparous WAP-T1 glands indicated that CK6a positive cells are present in luminal epithelia of ducts, but absent from hyperplasia composed of TAg positive cells (Figure 8A).There was no evidence for CK6a positive cells co-expressing TAg. In ducts, Ck6a positive luminal cells were often seen in close proximity to TAg expressing cells (Figure 8B). These data suggest that TAg does not target CK6a positive cells.

### TAg expressing cells are estrogen and progesterone receptor negative

**Figure 9:**
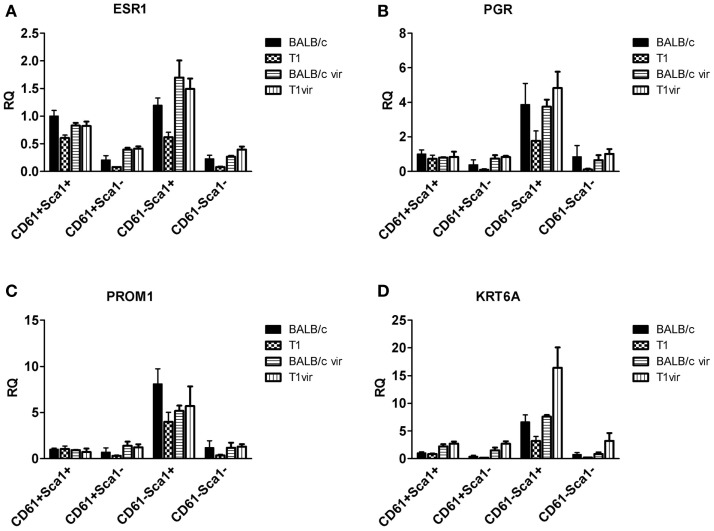
**qRT-PCR analysis of hormone receptor, prominin-1, and Ck6a expression in luminal cell subpopulations**. Expression of the estrogen receptor (ESR1) **(A)** and progesterone receptor (PGR) **(B)** in the CD61^−^Sca1^+^ ductal cell subpopulation coincides with expression of prominin-1 (PROM1) **(C)** and CK6a (KRT6A) **(D)**; subpopulations from virgin mice (BALB/c vir *n* = 3; T1vir *n* = 3); subpopulations from uniparous mice 120 days p.w. (BALB/c *n* = 4; T1 *n* = 5); data presented as mean ± SEM.

**Figure 10:**
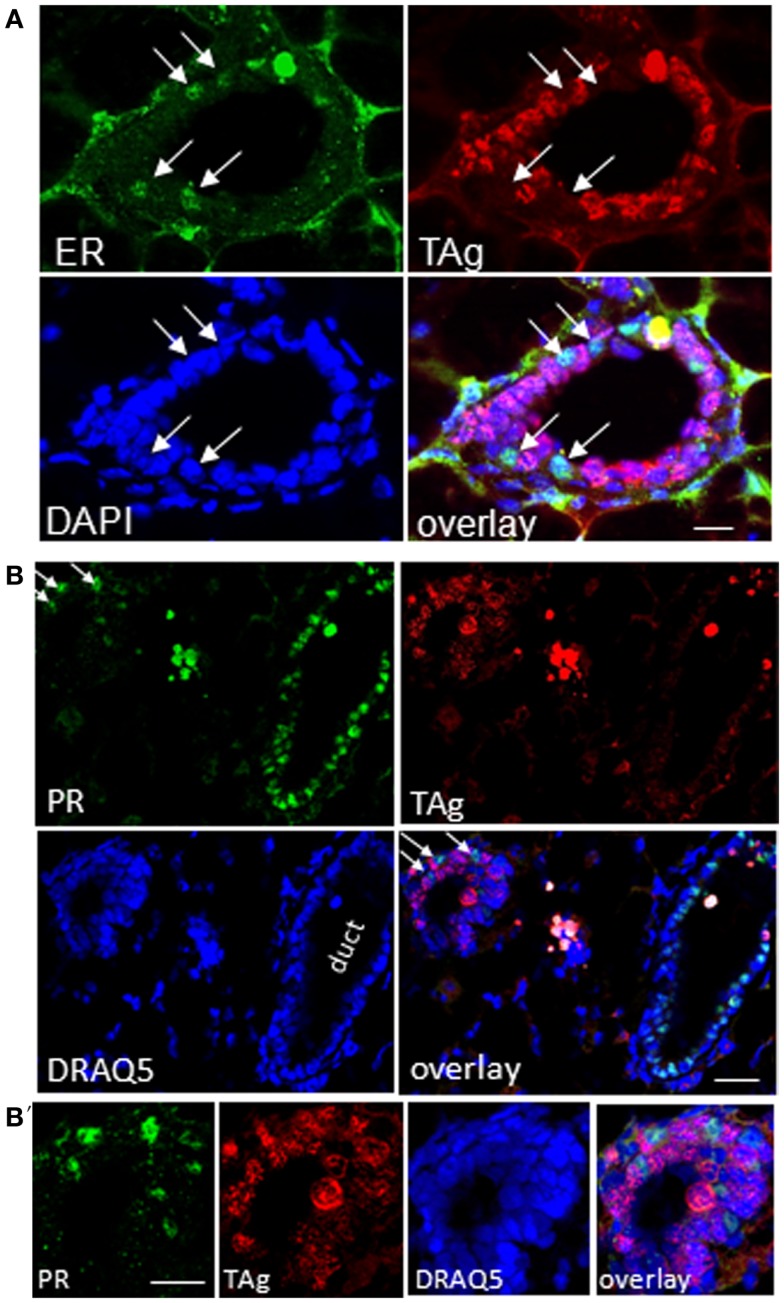
**TAg positive epithelia in resting uniparous WAP-T1 glands are negative for estrogen receptor (ER) and progesterone receptor (PR)**. **(A–B′)**: IF double labeling on cryosections from resting uniparous WAP-T1 glands; **(A)** Double labeling with antibodies to estrogen receptor (ER) (green) and TAg (red); staining of DNA with DAPI (blue); ER positive cells are TAg negative [arrows in **(A)**]; **(B,B′)**: double labeling with antibodies to progesterone receptor (PR) (green) and TAg (red); DNA staining with DAPI (blue); note the high number of PR positive but TAg negative cells in epithelia of ducts; no coincident labeling of PR [arrows in **(B)**] and TAg. Bars: **(A)** = 20 μm;**(B,B′)** = 50 μm.

It is well-known that proliferation and differentiation of luminal epithelia into lobulo-alveolar structures are under control of hormone receptors (38–41). Thus, we asked whether high *TAg* levels in luminal cell subpopulations derived from resting uniparous WAP-T1 glands correlated with high expression of either the estrogen (Esr1) or progesterone receptor (Pgr). qRT-PCR analysis showed prominent *Esr1* expression in Sca1^+^ subpopulations and a ~sevenfold lower expression in Sca1^−^ subpopulations from WAP-T1 (T1 in Figures 9A,B). A similar pattern was observed in subpopulations from resting uniparous BALB/c glands (BALB/c in Figures 9A,B). But, compared to WAP-T1, *Esr1* receptor expression was significantly higher (1.6-fold, *p* < 0.01 for CD61^+^/Sca1^+^; twofold, *p* < 0.01 for CD61^−^/Sca1^+^). Interestingly, WAP-T1 subpopulations with high *Esr1* levels showed low expression of *TAg*, *Elf5*, and milk genes (compare with Figure 7), suggesting that TAg and estrogen receptor mark different cells. In accordance by immunofluorescence, we found no overlap between estrogen receptor (ER) and TAg staining in luminal epithelia of glands (see Figure 10A).

*Pgr* expression levels in luminal cell subpopulations from BALB/c glands generally exceeded those in subpopulations from WAP-T1 glands (BALB/c and T1 in Figure 9B). *Pgr* levels were most prominent in subpopulations enriched in CD61^−^/Sca1^+^ cells with a tendency to be higher (~twofold, n.s.) in BALB/c compared to WAP-T1. Sca1^−^ subpopulations of BALB/c and WAP-T1 mice exhibited strongly reduced expression levels of *Pgr*. Thus, *Pgr* expression also shows a negative correlation with expression of *TAg*, *Elf5*, and milk genes as observed for *Esr1*. *In situ*, progesterone (PR) positive cells were present in luminal epithelia of ducts and generally showed no overlapping staining with antibodies to TAg (Figures 10B,B′). Taken together, these data indicate that TAg expressing luminal epithelial cells are estrogen and progesterone receptor negative.

### Hormone receptor positive cells localize to ductal epithelia and express CK6a, prominin-1, and Sca1

**Figure 11:**
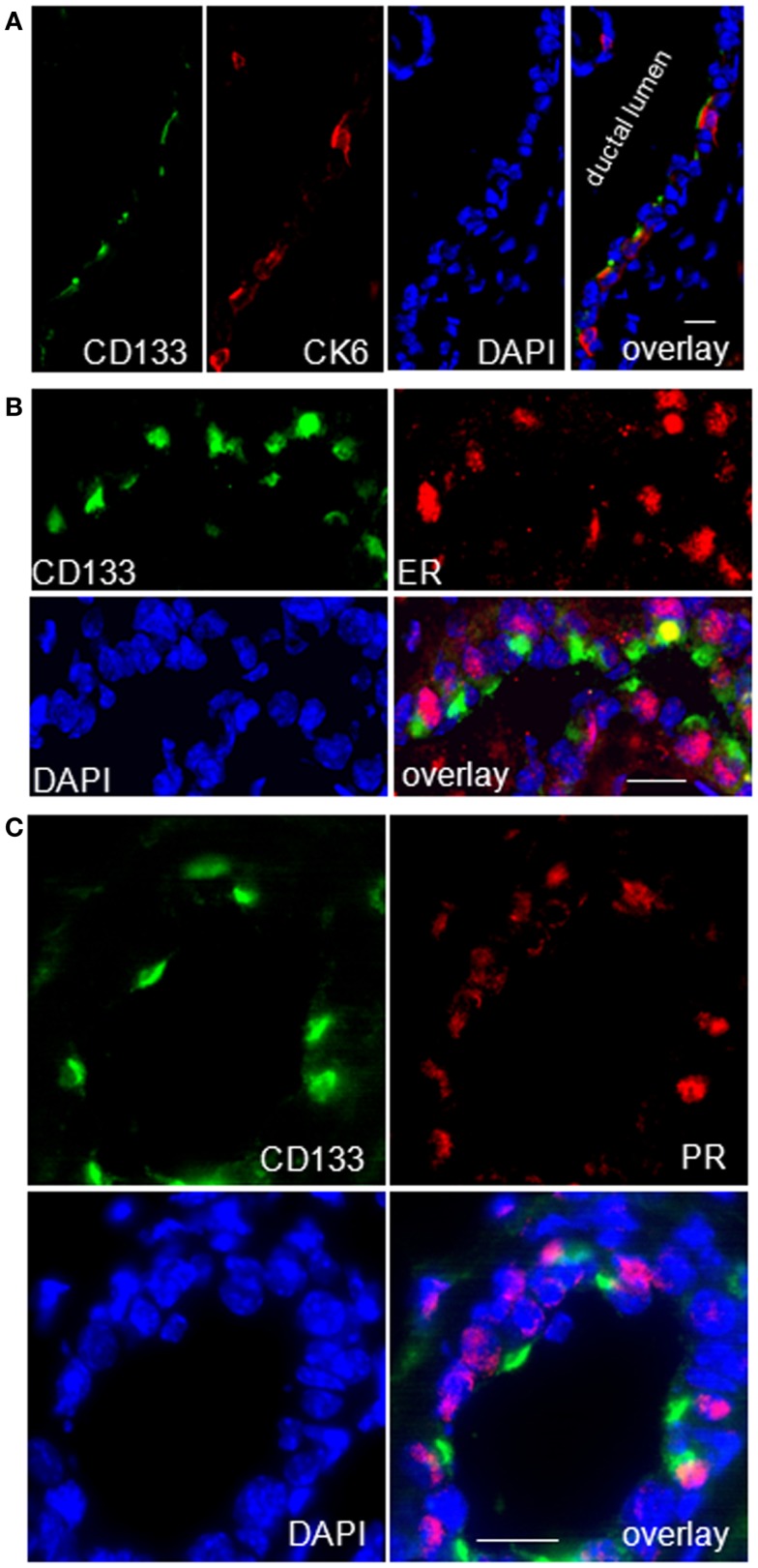
**CK6a marks CD133 (prominin-1) and hormone receptor positive cells in luminal epithelia of WAP-T1 glands**. **(A–C)** IF double labeling on cryosections of resting uniparous WAP-T1 glands (120 days p.w.); DNA staining with DAPI (blue); **(A)** Coincident staining of luminal epithelial cells in ducts with antibodies to CD133 (green) and CK6 (red); **(B)** Coincident labeling of luminal cells with antibodies to CD133 (green) and estrogen receptor (red); **(C)** Coincident staining of luminal epithelial cells with antibodies to CD133 and progesterone receptor (PR); note the cap-like staining of CD133 at the luminal side of epithelial cells in **(A–C)**. Bars: **(A)** = 20 μm;**(B,C)** = 50 μm.

Previous studies of others on adult virgin mice showed that estrogen and progesterone receptor expression are confined to “hormone sensing cells” located in the ductal epithelium; these cells were shown to express prominin-1 (CD133) and Sca1 and revealed a relatively differentiated phenotype (42). In line with these data, our qRT-PCR analysis showed high expression of *Prom1* specifically in the CD61^−^/Sca^+^ subpopulation both, from WAP-T1 and BALB/c, and this population also exhibited high expression levels of *Esr1* and *Pgr* (BALB/c and T1 in Figures 9C,D). *Krt6a* expression was also most prominent in this subpopulation. Therefore, we asked whether CK6a and prominin-1 localized to identical epithelial cells. IF studies performed on cryosections of resting uniparous WAP-T1 glands demonstrated that CD133 (prominin-1) and CK6a antibodies marked the same cells in luminal epithelia of ducts (Figure 11A). CD133 positive cells were also positive for the estrogen (Figure 11B), respectively progesterone receptor (Figure 11C), but definitely negative for TAg. Our data indicate that CK6a, prominin-1, and Sca1 are common markers of hormone receptor positive cells in luminal epithelia of ducts. These cells proved to be absent from hyperplasia in resting uniparous WAP-T1 glands, but in ducts they often localized in close proximity to TAg expressing cells.

### Resting glands of aged WAP-T1 virgin mice are not sensitized to alveologenesis

At this point, we asked whether WAP-T1 specific changes seen in resting glands of parous animals are already pre-determined at the virgin stage. We assumed that significant alterations should accumulate with time and thus be most prominent in aged virgin mice. Therefore, we extended our gene expression analysis to aged WAP-T1 and BALB/c virgin mice and isolated luminal cell subpopulations at 160 days post-partum (p.p.). We combined the qRT-PCR results shown in Figures 7 and 9 with the data obtained with resting glands. qRT-PCR of aged virgin mice showed low and nearly identical *Elf5* levels in Sca1^−^ subpopulations of both strains (see BALB/c vir and T1 vir in Figure 7A).The milk genes *Wap* and *Lalba* were barely expressed (see BALB/c vir and T1 vir in Figure 7B). *Esr1* and *Pgr* levels in luminal cell subpopulations of aged virgins were virtually identical in both mouse strains (see BALB/c vir and T1 vir in Figures 9A,B) and similar to the level in resting (120 days p.w.) glands of normal BALB/c mice (see BALB/c in Figures 9A,B). They reached the highest levels in CD61^−^/Sca1^+^ subpopulations, and like in resting glands these populations also showed highest expression of *Prom1* and *Krt6a* (BALB/c vir and T1 vir in Figures 9C,D).

These results demonstrate that glands of aged virgin mice display a hormone receptor status similar to that of resting glands. However, pathways leading to expression of ELF5 and milk genes seem not to be active at the virgin stage in both mouse strains. Therefore, changes observed in resting uniparous WAP-T1 glands are not already pre-determined in virgin mice. The data indicate that passage through pregnancy, lactation, and involution sensitizes luminal cell populations in resting glands of parous BALB/c and WAP-T1 mice to pathways of alveologenesis.

## Discussion

Our data show that hyperplasia composed of TAg expressing epithelial cells in resting uniparous WAP-T1 glands display features of lobulo-alveolar cells. The cells not only express ELF5, a transcription factor known to specify secretory alveolar cell fate of CD61^+^ precursors in the mature alveolar epithelium (43, 44), but are secretory as indicated by expression of milk genes. This suggests they reach an advanced stage in alveolar differentiation.

As shown by immunofluorescence, TAg positive cells in lactating WAP-T1 glands and in hyperplasia of resting uniparous WAP-T1 glands did not express Sca1; Sca1 positive cells were clearly confined to ductal epithelia. It suggests that Sca1 is a marker which separates ductal cells from cells with an alveolar cell fate in the mouse mammary gland. In accordance, *ELF5* and milk gene expression clearly separated with Sca1 negative subpopulations in our RT-PCR analysis.

*TAg* was expressed at a high level not only in CD61^−^/Sca1^−^, but also in CD61^+^/Sca1^−^ cells. This led us to assume that TAg targets luminal epithelial cells early during alveolar differentiation. Possibly, TAg expression is initiated with the onset of ELF5 expression, as this master regulator of alveologenesis was shown to induce WAP expression (43). Additional markers are needed to precisely decipher alveolar differentiation of luminal cells in relation to TAg expression. Progress is being made in this area as a recent study identified hitherto unknown epithelial cell lineages that regulate spatial placement of tertiary branches as well as formation of alveolar clusters in ducts of mammary glands (45). It would be interesting to see if the onset of TAg expression coincides with early priming to the alveolar lineage.

*Elf5* expression was not only seen in resting uniparous WAP-T1 glands, but also in resting uniparous BALB/c glands. We show that CD61^+^/Sca1^−^ subpopulations from WAP-T1 and BALB/c glands exhibited nearly identical, high levels of *Elf5*. Thus, alveologenesis seen in resting WAP-T1 glands is not specifically induced by TAg. Alveolar differentiation and formation of lobulo-alveolar structures are initiated regularly in rodent mammary glands during estrous cycle (46). Normally, these structures regress during diestrus. Thus, we assume that development of hyperplasia in WAP-T1 mice is initiated by mechanisms normally inducing transient alveologenesis in parous mice. Considering that ELF5 induces the *Wap* promoter (43), it is conceivable that ELF5 sustains TAg expression in differentiating alveolar cells. Although the cells show lactogenic activity, they seem not to reach a terminal stage of alveolar differentiation, as they stay in cell cycle which is indicated by positive reaction with antibodies to Mcm2 and Ki67. Both of these markers are not expressed in differentiated cells (33, 34).

It has been well-established that differentiation into alveolar, secretory active cells is regulated by a complex regulatory network comprising hormone receptors and transcription factors (43, 44, 47, 48). Apparently, estrogen (ER) and progesterone receptor (PR) positive cells compose a hormone sensing compartment within the luminal epithelium of ducts that induces differentiation and proliferation of hormone receptor negative cells through paracrine mechanisms. In addition, proliferation of hormone sensitive cells seems to be stimulated directly by progesterone and estrogen (42, 49). Recently, a relatively differentiated subpopulation of luminal cells has been identified in glands of adult virgin mice displaying the markers CD24^high^/Sca1^+^/prominin-1^+^(CD133)/CK18^+^ and expressing estrogen, progesterone, and prolactin receptors. These cells represent little stem cell activity and show no proliferative activity, suggesting they constitute a hormone sensitive compartment (42). Here, we demonstrate for WAP-T1 glands that these cells localize to luminal epithelia of ducts, are TAg negative, but in close proximity to TAg positive cells. Furthermore, we show for the first time that ductal cells positive for hormone receptors and prominin-1 (CD133) are identical with those expressing cytokeratin CK6a. CK6a has been discussed to mark a population of luminal mammary progenitor cells (30, 50, 51), which lack repopulation activity (37). A progenitor cell function is compatible with changes in the relative proportion of cells during mammary gland development. These cells are found at significant quantity in mammary ducts of virgin mice, reduced in number during pregnancy, apparently absent from epithelia of lactating glands, and reappear in resting glands after involution (37).

Our qRT-PCR data further corroborate a relationship between expression of *CK6a*, *prominin-1*, and hormone receptor genes. *Prom1* and *Krt6a* expression were prominent in the same Sca1 positive subpopulation of luminal cells. This subpopulation also proved to be unique with regard to expression of the progesterone receptor, a hormone receptor known to drive mammary secretory differentiation via induction of ELF5 in luminal progenitors (47). Thus, we speculate that CK6a^+^/prominin^+^/Sca1^+^/ER^+^/PR^+^ luminal cells have a unique function in induction of TAg and ELF5 leading to the formation of TAg positive, estrogen, and progesterone receptor negative hyperplasia. Crossing of WAP-T1 mice with conditional *progesterone receptor* knock-out mice would be an interesting approach to investigate this hypothesis by testing for the requirement of this hormone receptor for tumor formation.

Features described here for WAP-T1 mice may also apply to other transgenic mouse models of mammary carcinogenesis that display hyperplasia with an alveolar phenotype. Similar to WAP-T1, C3(1)/SV40-T transgenic mice showed TAg expression in terminal duct lobular units (TDLU), an increased number of TDLU proliferative lesions and side ducts, and at later stages expansion of cells into the ductal lumen with multistage progression to carcinoma (52). Mice transgenic for the polyomavirus middle T-antigen (PyV-mT) under control of the MMTV promoter showed focal pre-malignant lesions and an enhanced number of abortive side buds with lumen that was positive for milk proteins (WAP and OPN) (53). Mice transgenic for *Wnt-1*, *Int-2*, *Cyclin D*, or *TGF*α under control of mammary specific promoters (MMTV, WAP, ß-lactoglobulin) also developed alveolar hyperplasia (54–56). In MMTV-neu transgenic mice, parity induced epithelial cells endowed with the potential to differentiate into alveolar or ductal cells were identified as targets for induction of tumorigenesis (24). Thus, hormone dependent activation of an oncogene in differentiating luminal epithelial cells of the mammary gland could generally be a crucial step to induction of aberrant proliferative activity leading to the formation of hyperplastic lesions.

## Author Contributions

Timo Quante, Florian Wegwitz, Wolfgang Deppert, and Wolfgang Bohn conceived and designed the experiments. Timo Quante and Florian Wegwitz designed and performed FACS and qRT-PCR analysis; Julia Abe and Alessandra Rossi were responsible for animal work and immunofluorescence analysis; Timo Quante, Wolfgang Deppert, and Wolfgang Bohn wrote the paper. The authors read and approved the final manuscript.

## Conflict of Interest Statement

The authors declare that the research was conducted in the absence of any commercial or financial relationships that could be construed as a potential conflict of interest.

## Supplementary Material

The Supplementary Material for this article can be found online at http://www.frontiersin.org/Journal/10.3389/fonc.2014.00168/abstract

Table S1 **Primer XS**.

## Abbreviations

CSN2, casein; ER, estrogen receptor; IF, immunofluorescence; LALBA, lactalbumin; n.s., not significant; PR, progesterone receptor; SV40, Simian virus 40; Tag, T-antigen; WAP, whey acidic protein.

## References

[1] Medina D Biological and molecular characteristics of the premalignant mouse mammary gland. Biochim Biophys Acta (2002) 1603(1):1–9 10.1016/S0304-419X(02)00053-7 12242106

[2] Mastracci TL Boulos FI Andrulis IL Lam WL Genomics and premalignant breast lesions: clues to the development and progression of lobular breast cancer. Breast Cancer Res (2007) 9(6):215 10.1186/bcr1785 18036272 PMC2246168

[3] Sinn HP Elsawaf Z Helmchen B Aulmann S Early breast cancer precursor lesions: lessons learned from molecular and clinical studies. Breast Care (Basel) (2010) 5(4):218–26 10.1159/000319624 22590441 PMC3346166

[4] Costarelli L Campagna D Mauri M Fortunato L Intraductal proliferative lesions of the breast-terminology and biology matter: premalignant lesions or preinvasive cancer? Int J Surg Oncol (2012) 2012:501904 10.1155/2012/501904 22655184 PMC3357964

[5] Abdel-Fatah TM Powe DG Hodi Z Reis-Filho JS Lee AH Ellis IO Morphologic and molecular evolutionary pathways of low nuclear grade invasive breast cancers and their putative precursor lesions: further evidence to support the concept of low nuclear grade breast neoplasia family. Am J Surg Pathol (2008) 32(4):513–23 10.1097/PAS.0b013e318161d1a5 18223478

[6] Schulze-Garg C Lohler J Gocht A Deppert W A transgenic mouse model for the ductal carcinoma in situ (DCIS) of the mammary gland. Oncogene (2000) 19(8):1028–37 10.1038/sj.onc.1203281 10713686

[7] Klein A Guhl E Zollinger R Tzeng YJ Wessel R Hummel M Gene expression profiling: cell cycle deregulation and aneuploidy do not cause breast cancer formation in WAP-SVT/t transgenic animals. J Mol Med (Berl) (2005) 83(5):362–76 10.1007/s00109-004-0625-1 15662539

[8] Mietz JA Unger T Huibregtse JM Howley PM The transcriptional transactivation function of wild-type p53 is inhibited by SV40 large T-antigen and by HPV-16 E6 oncoprotein. EMBO J (1992) 11(13):5013–20 1464323 10.1002/j.1460-2075.1992.tb05608.x PMC556979

[9] Dyson N Buchkovich K Whyte P Harlow E The cellular 107K protein that binds to adenovirus E1A also associates with the large T antigens of SV40 and JC virus. Cell (1989) 58(2):249–55 10.1016/0092-8674(89)90839-8 2546678

[10] Pittius CW Sankaran L Topper YJ Hennighausen L Comparison of the regulation of the whey acidic protein gene with that of a hybrid gene containing the whey acidic protein gene promoter in transgenic mice. Mol Endocrinol (1988) 2(11):1027–32 10.1210/mend-2-11-1027 2464745

[11] Schoenenberger CA Zuk A Groner B Jones W Andres AC Induction of the endogenous whey acidic protein (WAP) gene and a WAP-myc hybrid gene in primary murine mammary organoids. Dev Biol (1990) 139(2):327–37 10.1016/0012-1606(90)90302-Y 2186946

[12] Doppler W Villunger A Jennewein P Brduscha K Groner B Ball RK Lactogenic hormone and cell type-specific control of the whey acidic protein gene promoter in transfected mouse cells. Mol Endocrinol (1991) 5(11):1624–32 10.1210/mend-5-11-1624 1685765

[13] Millot B Fontaine ML Thepot D Devinoy E A distal region, hypersensitive to DNase I, plays a key role in regulating rabbit whey acidic protein gene expression. Biochem J (2001) 359(Pt 3):557–65 10.1042/0264-6021:3590557 11672429 PMC1222176

[14] Mukhopadhyay SS Wyszomierski SL Gronostajski RM Rosen JM Differential interactions of specific nuclear factor I isoforms with the glucocorticoid receptor and STAT5 in the cooperative regulation of WAP gene transcription. Mol Cell Biol (2001) 21(20):6859–69 10.1128/MCB.21.20.6859-6869.2001 11564870 PMC99863

[15] Tzeng YJ Guhl E Graessmann M Graessmann A Breast cancer formation in transgenic animals induced by the whey acidic protein SV40 T antigen (WAP-SV-T) hybrid gene. Oncogene (1993) 8(7):1965–71 8390039

[16] Santarelli R Tzeng YJ Zimmermann C Guhl E Graessmann A SV40 T-antigen induces breast cancer formation with a high efficiency in lactating and virgin WAP-SV-T transgenic animals but with a low efficiency in ovariectomized animals. Oncogene (1996) 12(3):495–505 8637705

[17] Li M Lewis B Capuco AV Laucirica R Furth PA WAP-TAg transgenic mice and the study of dysregulated cell survival, proliferation, and mutation during breast carcinogenesis. Oncogene (2000) 19(8):1010–9 10.1038/sj.onc.1203271 10713684

[18] Deeb KK Michalowska AM Yoon CY Krummey SM Hoenerhoff MJ Kavanaugh C Identification of an integrated SV40 T/t-antigen cancer signature in aggressive human breast, prostate, and lung carcinomas with poor prognosis. Cancer Res (2007) 67(17):8065–80 10.1158/0008-5472.CAN-07-1515 17804718

[19] Otto B Streichert T Wegwitz F Gevensleben H Klätschke K Wagener C Transcription factors link mouse WAP-T mammary tumors with human breast cancer. Int J Cancer (2013) 132:1311–22 10.1002/ijc.27941 23161608

[20] Bu W Chen J Morrison GD Huang S Creighton CJ Huang J Keratin 6A marks mammary bipotential progenitor cells that can give rise to a unique tumor model resembling human normal-like breast cancer. Oncogene (2011) 30(43):4399–409 10.1038/onc.2011.147 21532625 PMC3156856

[21] Boecker W Moll R Dervan P Buerger H Poremba C Diallo RI Usual ductal hyperplasia of the breast is a committed stem (progenitor) cell lesion distinct from atypical ductal hyperplasia and ductal carcinoma in situ. J Pathol (2002) 198(4):458–67 10.1002/path.1241 12434415

[22] Li Y Welm B Podsypanina K Huang S Chamorro M Zhang X Evidence that transgenes encoding components of the Wnt signaling pathway preferentially induce mammary cancers from progenitor cells. Proc Natl Acad Sci U S A (2003) 100(26):15853–8 10.1073/pnas.2136825100 14668450 PMC307657

[23] Liu BY McDermott SP Khwaja SS Alexander CM The transforming activity of Wnt effectors correlates with their ability to induce the accumulation of mammary progenitor cells. Proc Natl Acad Sci U S A (2004) 101(12):4158–63 10.1073/pnas.0400699101 15020770 PMC384711

[24] Henry MD Triplett AA Oh KB Smith GH Wagner KU Parity-induced mammary epithelial cells facilitate tumorigenesis in MMTV-neu transgenic mice. Oncogene (2004) 23(41):6980–5 10.1038/sj.onc.1207827 15286714

[25] Visvader JE Keeping abreast of the mammary epithelial hierarchy and breast tumorigenesis. Genes Dev (2009) 23(22):2563–77 10.1101/gad.1849509 19933147 PMC2779757

[26] Otto B Gruner K Heinlein CH Wegwitz F Nollau P Ylstra B Low-grade and high-grade mammary carcinomas in WAP-T transgenic mice are independent entities distinguished by Met expression. Int J Cancer (2013) 132:1300–10 10.1002/ijc.27783 22907219

[27] Wegwitz F Kluth MA Mänz C Otto B Gruner K Heinlein C Tumorigenic WAP-T mouse mammary carcinoma cells: a model for a self-reproducing homeostatic cancer cell system. PLoS One (2010) 5(8):e12103 10.1371/journal.pone.0012103 20730114 PMC2920333

[28] Welm BE Tepera SB Venezia T Graubert TA Rosen JM Goodell MA Sca-1(pos) cells in the mouse mammary gland represent an enriched progenitor cell population. Dev Biol (2002) 245(1):42–56 10.1006/dbio.2002.0625 11969254

[29] Shackleton M Vaillant F Simpson KJ Stingl J Smyth GK Asselin-Labat ML Generation of a functional mammary gland from a single stem cell. Nature (2006) 439(7072):84–8 10.1038/nature04372 16397499

[30] Stingl J Eirew P Ricketson I Shackleton M Vaillant F Choi D Purification and unique properties of mammary epithelial stem cells. Nature (2006) 439(7079):993–7 10.1038/nature04496 16395311

[31] Lee HJ Ormandy CJ Elf5, hormones and cell fate. Trends Endocrinol Metab (2012) 23(6):292–8 10.1016/j.tem.2012.02.006 22464677

[32] Dudderidge TJ Stoeber K Loddo M Atkinson G Fanshawe T Griffiths DF Mcm2, Geminin, and Ki67 define proliferative state and are prognostic markers in renal cell carcinoma. Clin Cancer Res (2005) 11(7):2510–7 10.1158/1078-0432.CCR-04-1776 15814627

[33] Shetty A Loddo M Fanshawe T Prevost AT Sainsbury R Williams GH DNA replication licensing and cell cycle kinetics of normal and neoplastic breast. Br J Cancer (2005) 93(11):1295–300 10.1038/sj.bjc.6602829 16278669 PMC2361513

[34] Scholz T Gerdes J The Ki-67 protein: from the known and the unknown. J Cell Physiol (2000) 182(3):311–22 10.1002/(SICI)1097-4652(200003)182:3<311::AID-JCP1>3.0.CO;2-9 10653597

[35] Asselin-Labat ML Shackleton M Stingl J Vaillant F Forrest NC Eaves CJ Steroid hormone receptor status of mouse mammary stem cells. J Natl Cancer Inst (2006) 98(14):1011–4 10.1093/jnci/djj267 16849684

[36] Shehata M Teschendorff A Sharp G Novcic N Russell IA Avril S Phenotypic and functional characterisation of the luminal cell hierarchy of the mammary gland. Breast Cancer Res (2012) 14(5):R134 10.1186/bcr3334 23088371 PMC4053112

[37] Sun P Yuan Y Li A Li B Dai X Cytokeratin expression during mouse embryonic and early postnatal mammary gland development. Histochem Cell Biol (2010) 133(2):213–21 10.1007/s00418-009-0662-5 19937336 PMC2807942

[38] Brisken C Park S Vass T Lydon JP O’Malley BW Weinberg RA A paracrine role for the epithelial progesterone receptor in mammary gland development. Proc Natl Acad Sci U S A (1998) 95(9):5076–81 10.1073/pnas.95.9.5076 9560231 PMC20216

[39] Lydon JP DeMayo FJ Funk CR Mani SK Hughes AR Montgomery CA Jr Mice lacking progesterone receptor exhibit pleiotropic reproductive abnormalities. Genes Dev (1995) 9(18):2266–78 10.1101/gad.9.18.2266 7557380

[40] Clarke CL Sutherland RL Progestin regulation of cellular proliferation. Endocr Rev (1990) 11(2):266–301 10.1210/edrv-11-2-266 2114281

[41] Atwood CS Hovey RC Glover JP Chepko G Ginsburg E Robison WG Progesterone induces side-branching of the ductal epithelium in the mammary glands of peripubertal mice. J Endocrinol (2000) 167(1):39–52 10.1677/joe.0.1670039 11018751

[42] Sleeman KE Kendrick H Robertson D Isacke CM Ashworth A Smalley MJ Dissociation of estrogen receptor expression and in vivo stem cell activity in the mammary gland. J Cell Biol (2007) 176(1):19–26 10.1083/jcb.200604065 17190790 PMC2063618

[43] Oakes SR Naylor MJ Asselin-Labat ML Blazek KD Gardiner-Garden M Hilton HN The Ets transcription factor Elf5 specifies mammary alveolar cell fate. Genes Dev (2008) 22(5):581–6 10.1101/gad.1614608 18316476 PMC2259028

[44] Asselin-Labat ML Mammary stem and progenitor cells: critical role of the transcription factor Gata-3. Med Sci (Paris) (2007) 23(12):1077–80 10.1051/medsci/200723121077 18154704

[45] Sale S Lafkas D Artavanis-Tsakonas S Notch2 genetic fate mapping reveals two previously unrecognized mammary epithelial lineages. Nat Cell Biol (2013) 15(5):451–60 10.1038/ncb2725 23604318 PMC4369920

[46] Schedin P Mitrenga T Kaeck M Estrous cycle regulation of mammary epithelial cell proliferation, differentiation, and death in the Sprague-Dawley rat: a model for investigating the role of estrous cycling in mammary carcinogenesis. J Mammary Gland Biol Neoplasia (2000) 5(2):211–25 10.1023/A:1026447506666 11149574

[47] Lee HJ Gallego-Ortega D Ledger A Schramek D Joshi P Szwarc MM Progesterone drives mammary secretory differentiation via RankL-mediated induction of Elf5 in luminal progenitor cells. Development (2013) 140(7):1397–401 10.1242/dev.088948 23462470

[48] Obr AE Edwards DP The biology of progesterone receptor in the normal mammary gland and in breast cancer. Mol Cell Endocrinol (2012) 357(1–2):4–17 10.1016/j.mce.2011.10.030 22193050 PMC3318965

[49] Beleut M Rajaram RD Caikovski M Ayyanan A Germano D Choi Y Two distinct mechanisms underlie progesterone-induced proliferation in the mammary gland. Proc Natl Acad Sci U S A (2010) 107(7):2989–94 10.1073/pnas.0915148107 20133621 PMC2840294

[50] Smith GH Mehrel T Roop DR Differential keratin gene expression in developing, differentiating, preneoplastic, and neoplastic mouse mammary epithelium. Cell Growth Differ (1990) 1(4):161–70 1707299

[51] Grimm SL Bu W Longley MA Roop DR Li Y Rosen JM Keratin 6 is not essential for mammary gland development. Breast Cancer Res (2006) 8(3):R29 10.1186/bcr1584 16790075 PMC1557733

[52] Green JE Shibata MA Yoshidome K Liu ML Jorcyk C Anver MR The C3(1)/SV40 T-antigen transgenic mouse model of mammary cancer: ductal epithelial cell targeting with multistage progression to carcinoma. Oncogene (2000) 19(8):1020–7 10.1038/sj.onc.1203280 10713685

[53] Maglione JE Moghanaki D Young LJ Manner CK Ellies LG Joseph SO Transgenic Polyoma middle-T mice model premalignant mammary disease. Cancer Res (2001) 61(22):8298–305 11719463

[54] Tsukamoto AS Grosschedl R Guzman RC Parslow T Varmus HE Expression of the int-1 gene in transgenic mice is associated with mammary gland hyperplasia and adenocarcinomas in male and female mice. Cell (1988) 55(4):619–25 10.1016/0092-8674(88)90220-6 3180222

[55] Wang TC Cardiff RD Zukerberg L Lees E Arnold A Schmidt EV Mammary hyperplasia and carcinoma in MMTV-cyclin D1 transgenic mice. Nature (1994) 369(6482):669–71 10.1038/369669a0 8208295

[56] Sandgren EP Luetteke NC Palmiter RD Brinster RL Lee DC Overexpression of TGF alpha in transgenic mice: induction of epithelial hyperplasia, pancreatic metaplasia, and carcinoma of the breast. Cell (1990) 61(6):1121–35 10.1016/0092-8674(90)90075-P 1693546

